# Crystal structure of bis­(4-meth­oxy­phenyl) malonate

**DOI:** 10.1107/S2056989015006891

**Published:** 2015-04-22

**Authors:** H. C. Devarajegowda, P. A. Suchetan, S. Sreenivasa, H. T. Srinivasa, B. S. Palakshamurthy

**Affiliations:** a Department of Physics, Yuvaraja’s College (Constituent College), University of, Mysore, Mysore, Karnataka 570 005, India; b Department of Studies and Research in Chemistry, U.C.S., Tumkur University, Tumkur, Karnataka, 572 103, India; c Department of Studies and Research in Chemistry, Tumkur University, Tumkur, Karnataka 572 103, India; d Raman Research Institute, C. V. Raman, Avenue, Sadashivanagar, Bangalore, Karnataka, India

**Keywords:** crystal structure, orientational disorder, C—H⋯O inter­actions, C—H⋯π inter­actions

## Abstract

The complete mol­ecule of the title compound, C_17_H_16_O_6_, is generated by crystallographic twofold symmetry, with the central methyl­ene C atom lying on the rotation axis. The carbonyl O atom is disordered over two adjacent positions in a 0.63 (3):0.37 (3) ratio and the dihedral angle between the benzene rings in the two halves of the mol­ecule is 79.31 (12)°. In the crystal, mol­ecules are connected by C—H⋯O hydrogen bonds, generating (110) sheets. Very weak intra­sheet C—H⋯π inter­actions are also observed.

## Related literature   

For the application of the 4-meth­oxy­phenyl group in chemiluminescence, see: Teranishi *et al.*(1999 ▸). For its biological activity, see: Prasanna Kumar *et al.*, (2013 ▸).
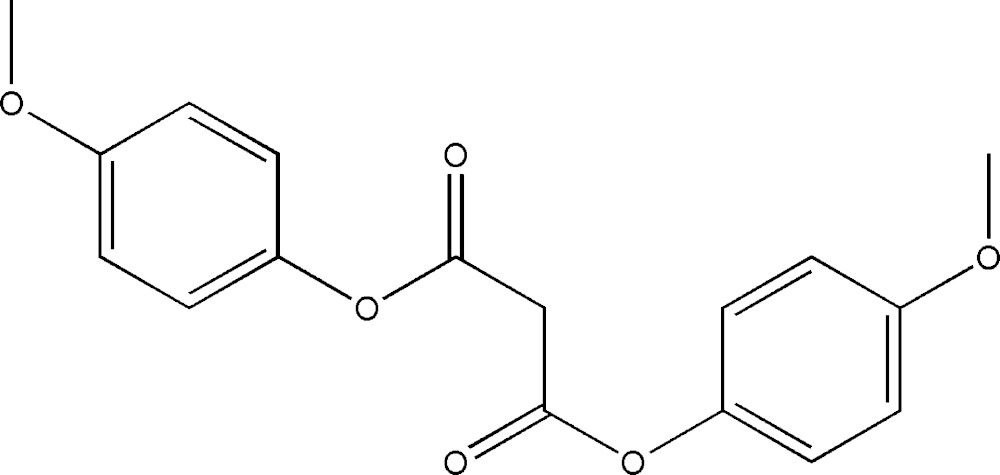


## Experimental   

### Crystal data  

C_17_H_16_O_6_
*M* *_r_* = 316.30
Orthorhombic, 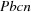

*a* = 5.4307 (19) Å
*b* = 8.131 (3) Å
*c* = 36.149 (10) Å
*V* = 1596.3 (9) Å^3^
*Z* = 4
Mo *K*α radiation
μ = 0.10 mm^−1^
*T* = 296 K
0.18 × 0.16 × 0.14 mm

### Data collection  

Bruker APEXII CCD diffractometer
Absorption correction: multi-scan (*SADABS*; Bruker, 2013 ▸) *T*_min_ = 0.982, *T*_max_ = 0.986
6486 measured reflections
1405 independent reflections
1008 reflections with *I* > 2σ(*I*)
*R*_int_ = 0.036

### Refinement  

*R*[*F* ^2^ > 2σ(*F* ^2^)] = 0.041
*wR*(*F* ^2^) = 0.162
*S* = 1.03
1405 reflections
121 parameters
6 restraints
H atoms treated by a mixture of independent and constrained refinement
Δρ_max_ = 0.18 e Å^−3^
Δρ_min_ = −0.16 e Å^−3^

### 

Data collection: *APEX2* (Bruker, 2013 ▸); cell refinement: *SAINT* (Bruker, 2013 ▸); data reduction: *SAINT*; program(s) used to solve structure: *SHELXS97* (Sheldrick, 2008 ▸); program(s) used to refine structure: *SHELXL2014*/7 (Sheldrick, 2015 ▸); molecular graphics: *ORTEP-3 for Windows* (Farrugia, 2012 ▸) and *Mercury* (Macrae *et al.*, 2008 ▸); software used to prepare material for publication: *SHELXL97* (Sheldrick, 2008 ▸).

## Supplementary Material

Crystal structure: contains datablock(s) I, New_Global_Publ_Block. DOI: 10.1107/S2056989015006891/hb7398sup1.cif

Structure factors: contains datablock(s) I. DOI: 10.1107/S2056989015006891/hb7398Isup2.hkl

**Fig. 1.**
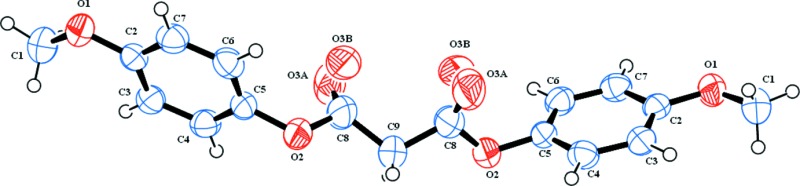
The molecular structure of the title compound, showing displacement ellipsoids drawn at the 50% probability level.

**Fig. 2.**
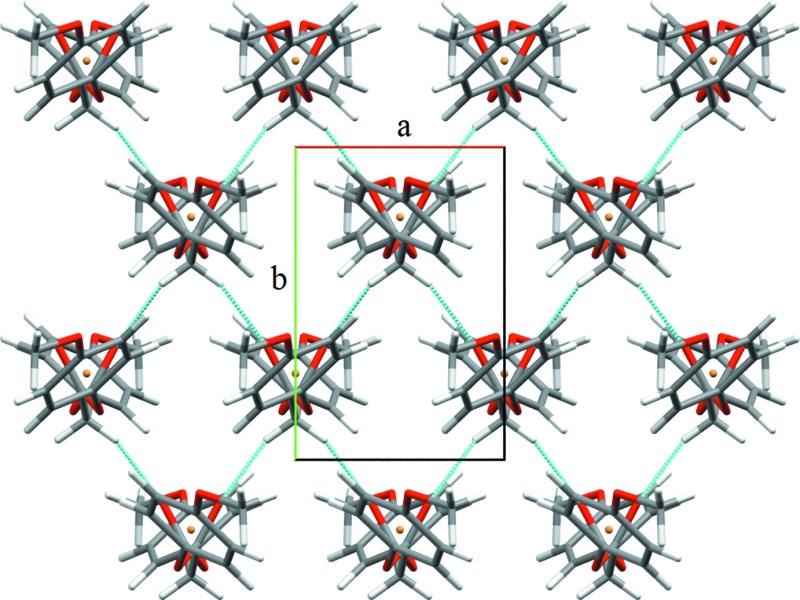
The molecular packing of the title compound when viewed along c axis. Dashed lines indicate intermolecular C—H···O interactions.

**Fig. 3.**
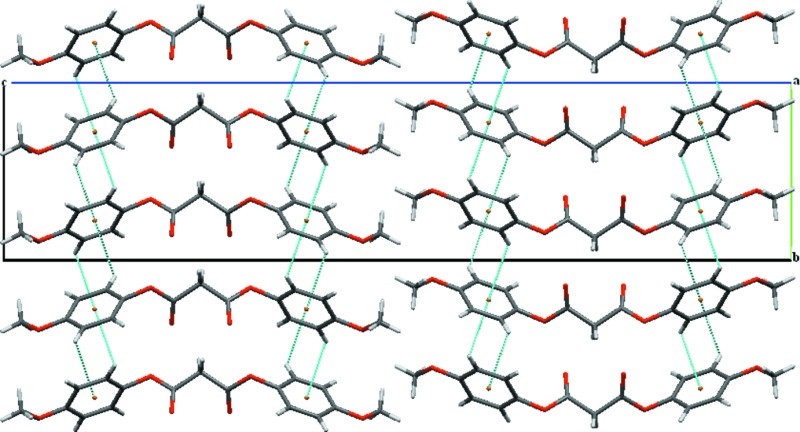
The molecular packing of the title compound when viewed along a axis. Dashed lines indicate intermolecular C—H···π interactions.

. DOI: 10.1107/S2056989015006891/hb7398fig1.tif
The mol­ecular structure of the title compound, showing displacement ellipsoids drawn at the 50% probability level.

c . DOI: 10.1107/S2056989015006891/hb7398fig2.tif
The mol­ecular packing of the title compound when viewed along *c* axis. Dashed lines indicate inter­molecular C—H⋯O inter­actions.

a . DOI: 10.1107/S2056989015006891/hb7398fig3.tif
The mol­ecular packing of the title compound when viewed along *a* axis. Dashed lines indicate inter­molecular C—H⋯π inter­actions.

CCDC reference: 1058073

Additional supporting information:  crystallographic information; 3D view; checkCIF report

## Figures and Tables

**Table 1:** Hydrogen-bond geometry (, ) *Cg*1 is the centroid of the benzene ring.

*D*H*A*	*D*H	H*A*	*D* *A*	*D*H*A*
C9H9*A*O3*A* ^i^	0.92(3)	2.53(3)	3.216(6)	131(3)
C4H4*Cg*1^ii^	0.93	2.99	3.6957	134
C7H7*Cg*1^iii^	0.93	2.99	3.6980	134

Symmetry codes: (i) 
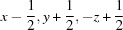
; (ii) 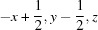; (iii) 
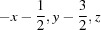
.

## supplementary crystallographic information

### S1. Chemical context

4-Meth­oxy­phenyl derivatives play significant role in synthesizing chemiluminescence (Teranishi *et al.*, 1999), biologically active materials (Prasanna Kumar *et al.*, 2013) and molecule-based magnetic materials etc., Keeping these things in mind, and our inter­est towards synthesizing liquid crystals bearing malonate moiety [–C(O)O—CH2—C(O)O-], we report here the crystal structure of the title compound.

### S2. Structural commentary

The molecules of the title compound, C_17_H_16_O_6_, show two fold rotation symmetry, for which the 2-fold rotation crystallographic axis passes through the C9 atom (with symmetry code -*x*, *y*, -*z*+1/2). The asymmetric unit of the title compound contains half molecule. The carbonyl oxygen atom is disordered over two positions due to crystallographic 2-fold rotation axis (orientational disorder), the occupancy ratio being 0.63 (3) : 0.37 (3). The dihedral angle between the benzene rings in the two halves of the molecule is 79.31 (12)^o^. Further, the dihedral angle between the central –CH_2_–C(O)–O– segment and the phenyl ring is 86.41 (6)^o^. The meth­oxy group is approximately coplanar with the attached benzene ring, the C1—O1—C2—C3 torsion being 3.76 (1)^o^ .

### S3. Supra­molecular features

In the crystal structure, the molecules are connected via C9—H9···O3 inter­molecular inter­actions running into C(4) chains along crystallographic a and b axis, thus forming sheets in the ab plane. These sheets are further stabilized by C4—H4···pi and C7—H7···pi inter­actions (where Cg is the centroid of the phenyl ring)along [010], and thus, a two dimensional architecture is observed.

### S4. Synthesis and crystallization

A mixture of malonic acid (1 mmol) and phospho­rous oxychloride (POCl_3_) was stirred for about an hour at 30°C . To this mixture, 4-meth­oxy­phenol (2 mmol) was added and the reaction mixture was heated to 50°C for 30 minutes. The reaction mixture was poured into crushed ice and the solid obtained was thoroughly washed with water, dilute sodium hydroxide and again with water.

Colourless blocks of the title compound were obtained from slow evaporation technique using methanol as the solvent.

### S5. Refinement details

Crystal data, data collection and structure refinement details are summarized in Table 1. The H atoms were positioned with idealized geometry using a riding model with C—H = 0.95-0.99 Å. All H-atoms were refined with isotropic displacement parameters (set to 1.2-1.5 times of the U eq of the parent atom). The carbonyl oxygen atom is disordered over two sites and refined with site occupancy factors 0.63 (3) : 0.37 (3).

### Crystal data

**Table:**

C_17_H_16_O_6_	Block
*M**_r_* = 316.30	*D*_x_ = 1.316 Mg m^−^^3^
Orthorhombic, *P**b**c**n*	Melting point: 465 K
Hall symbol: -P 2n 2ab	Mo *K*α radiation, λ = 0.71073 Å
*a* = 5.4307 (19) Å	Cell parameters from 1405 reflections
*b* = 8.131 (3) Å	θ = 3.4–25.0°
*c* = 36.149 (10) Å	µ = 0.10 mm^−^^1^
*V* = 1596.3 (9) Å^3^	*T* = 296 K
*Z* = 4	Block, colourless
*F*(000) = 664	0.18 × 0.16 × 0.14 mm

### Data collection

**Table:**

Bruker APEXII CCD diffractometer	1405 independent reflections
Radiation source: fine-focus sealed tube	1008 reflections with *I* > 2σ(*I*)
Graphite monochromator	*R*_int_ = 0.036
Detector resolution: 2.09 pixels mm^-1^	θ_max_ = 25.0°, θ_min_ = 3.4°
phi and ω scans	*h* = −6→6
Absorption correction: multi-scan (*SADABS*; Bruker, 2013)	*k* = −9→8
*T*_min_ = 0.982, *T*_max_ = 0.986	*l* = −42→42
6486 measured reflections	

### Refinement

**Table:**

Refinement on *F*^2^	Secondary atom site location: difference Fourier map
Least-squares matrix: full	Hydrogen site location: mixed
*R*[*F*^2^ > 2σ(*F*^2^)] = 0.041	H atoms treated by a mixture of independent and constrained refinement
*wR*(*F*^2^) = 0.162	*w* = 1/[σ^2^(*F*_o_^2^) + (0.1061*P*)^2^ + 0.0364*P*] where *P* = (*F*_o_^2^ + 2*F*_c_^2^)/3
*S* = 1.03	(Δ/σ)_max_ = 0.004
1405 reflections	Δρ_max_ = 0.18 e Å^−^^3^
121 parameters	Δρ_min_ = −0.16 e Å^−^^3^
6 restraints	Extinction correction: *SHELXL2014*/7 (Sheldrick 2014, Fc^*^=kFc[1+0.001xFc^2^λ^3^/sin(2θ)]^-1/4^
2 constraints	Extinction coefficient: 0.019 (4)
Primary atom site location: structure-invariant direct methods	

### Special details

**Table:**

Geometry. All e.s.d.'s (except the e.s.d. in the dihedral angle between two l.s. planes) are estimated using the full covariance matrix. The cell e.s.d.'s are taken into account individually in the estimation of e.s.d.'s in distances, angles and torsion angles; correlations between e.s.d.'s in cell parameters are only used when they are defined by crystal symmetry. An approximate (isotropic) treatment of cell e.s.d.'s is used for estimating e.s.d.'s involving l.s. planes.

### Fractional atomic coordinates and isotropic or equivalent isotropic displacement parameters (Å^2^)

**Table:**

	*x*	*y*	*z*	*U*_iso_*/*U*_eq_	Occ. (<1)
O2	−0.0641 (3)	0.84493 (15)	0.31318 (4)	0.0610 (5)	
O1	0.0400 (3)	0.60669 (15)	0.45593 (4)	0.0591 (5)	
C8	0.0313 (6)	0.7692 (3)	0.28435 (6)	0.0854 (9)	
C1	0.2471 (4)	0.6502 (3)	0.47762 (6)	0.0697 (7)	
H1B	0.3947	0.6145	0.4654	0.105*	
H1A	0.2353	0.5983	0.5014	0.105*	
H1C	0.2515	0.7675	0.4807	0.105*	
C9	0.0000	0.8706 (5)	0.2500	0.0964 (15)	
C2	0.0277 (3)	0.66889 (19)	0.42034 (5)	0.0454 (5)	
C5	−0.0275 (3)	0.7785 (2)	0.34879 (5)	0.0468 (5)	
C4	0.1692 (3)	0.8324 (2)	0.36942 (5)	0.0511 (6)	
H4	0.2824	0.9052	0.3592	0.061*	
C7	−0.1689 (3)	0.6158 (2)	0.39913 (6)	0.0523 (5)	
H7	−0.2829	0.5430	0.4091	0.063*	
C6	−0.1967 (3)	0.6706 (2)	0.36313 (6)	0.0525 (6)	
H6	−0.3286	0.6348	0.3488	0.063*	
C3	0.1984 (3)	0.7778 (2)	0.40568 (5)	0.0499 (6)	
H3	0.3306	0.8138	0.4199	0.060*	
O3B	0.051 (3)	0.6253 (9)	0.2836 (3)	0.080 (4)	0.37 (3)
O3A	0.194 (5)	0.661 (2)	0.2893 (2)	0.152 (6)	0.63 (3)
H9A	−0.139 (6)	0.936 (4)	0.2521 (11)	0.150 (13)*	

### Atomic displacement parameters (Å^2^)

**Table:**

	*U*^11^	*U*^22^	*U*^33^	*U*^12^	*U*^13^	*U*^23^
O2	0.0850 (11)	0.0534 (8)	0.0445 (10)	0.0136 (6)	−0.0043 (7)	0.0011 (6)
O1	0.0721 (10)	0.0615 (9)	0.0438 (9)	−0.0056 (6)	0.0010 (6)	0.0048 (6)
C8	0.147 (3)	0.0634 (16)	0.0460 (15)	0.0256 (15)	0.0084 (14)	−0.0017 (11)
C1	0.0823 (16)	0.0756 (14)	0.0514 (14)	0.0012 (11)	−0.0106 (12)	0.0041 (10)
C9	0.183 (5)	0.062 (2)	0.045 (2)	0.000	−0.010 (2)	0.000
C2	0.0547 (12)	0.0396 (9)	0.0418 (12)	0.0040 (7)	0.0041 (8)	−0.0033 (7)
C5	0.0602 (12)	0.0424 (10)	0.0380 (11)	0.0083 (8)	−0.0005 (8)	−0.0023 (7)
C4	0.0547 (12)	0.0448 (10)	0.0538 (13)	−0.0037 (7)	0.0023 (9)	0.0015 (8)
C7	0.0523 (12)	0.0483 (10)	0.0562 (13)	−0.0066 (8)	0.0061 (9)	−0.0019 (8)
C6	0.0509 (12)	0.0514 (11)	0.0553 (13)	−0.0006 (8)	−0.0072 (9)	−0.0071 (9)
C3	0.0529 (12)	0.0471 (10)	0.0497 (12)	−0.0045 (8)	−0.0054 (8)	−0.0016 (8)
O3B	0.140 (8)	0.043 (5)	0.057 (4)	0.026 (4)	0.001 (4)	−0.006 (2)
O3A	0.244 (14)	0.158 (7)	0.053 (3)	0.132 (9)	0.017 (5)	0.002 (3)

### Geometric parameters (Å, º)

**Table:**

O2—C8	1.316 (3)	C9—H9A	0.92 (3)
O2—C5	1.410 (2)	C2—C7	1.383 (3)
O1—C2	1.384 (2)	C2—C3	1.387 (3)
O1—C1	1.416 (3)	C5—C6	1.372 (3)
C8—O3B	1.176 (9)	C5—C4	1.375 (3)
C8—O3A	1.262 (10)	C4—C3	1.393 (3)
C8—C9	1.500 (3)	C4—H4	0.9300
C1—H1B	0.9600	C7—C6	1.384 (3)
C1—H1A	0.9600	C7—H7	0.9300
C1—H1C	0.9600	C6—H6	0.9300
C9—C8^i^	1.500 (3)	C3—H3	0.9300
			
C8—O2—C5	119.22 (16)	O1—C2—C3	123.81 (16)
C2—O1—C1	117.48 (15)	C7—C2—C3	120.20 (18)
O3B—C8—O2	121.3 (5)	C6—C5—C4	121.32 (17)
O3A—C8—O2	119.4 (4)	C6—C5—O2	119.70 (16)
O3B—C8—C9	122.6 (6)	C4—C5—O2	118.82 (16)
O3A—C8—C9	125.5 (5)	C5—C4—C3	119.79 (17)
O2—C8—C9	110.7 (2)	C5—C4—H4	120.1
O1—C1—H1B	109.5	C3—C4—H4	120.1
O1—C1—H1A	109.5	C2—C7—C6	120.35 (17)
H1B—C1—H1A	109.5	C2—C7—H7	119.8
O1—C1—H1C	109.5	C6—C7—H7	119.8
H1B—C1—H1C	109.5	C5—C6—C7	119.19 (17)
H1A—C1—H1C	109.5	C5—C6—H6	120.4
C8—C9—C8^i^	113.4 (3)	C7—C6—H6	120.4
C8—C9—H9A	110 (2)	C2—C3—C4	119.15 (17)
C8^i^—C9—H9A	107 (2)	C2—C3—H3	120.4
O1—C2—C7	116.00 (16)	C4—C3—H3	120.4
			
C5—O2—C8—O3B	32.7 (11)	C6—C5—C4—C3	−0.3 (3)
C5—O2—C8—O3A	−14.9 (17)	O2—C5—C4—C3	175.19 (14)
C5—O2—C8—C9	−172.58 (17)	O1—C2—C7—C6	−179.78 (15)
O3B—C8—C9—C8^i^	6.5 (10)	C3—C2—C7—C6	0.1 (3)
O3A—C8—C9—C8^i^	56.1 (17)	C4—C5—C6—C7	0.3 (3)
O2—C8—C9—C8^i^	−147.9 (3)	O2—C5—C6—C7	−175.16 (15)
C1—O1—C2—C7	176.14 (16)	C2—C7—C6—C5	−0.2 (3)
C1—O1—C2—C3	−3.8 (3)	O1—C2—C3—C4	179.77 (16)
C8—O2—C5—C6	−91.6 (2)	C7—C2—C3—C4	−0.1 (3)
C8—O2—C5—C4	92.8 (2)	C5—C4—C3—C2	0.2 (3)

Symmetry code: (i) −*x*, *y*, −*z*+1/2.

### Hydrogen-bond geometry (Å, º)

Cg1 is the centroid of the benzene ring.

**Table:**

*D*—H···*A*	*D*—H	H···*A*	*D*···*A*	*D*—H···*A*
C9—H9*A*···O3*A*^ii^	0.92 (3)	2.53 (3)	3.216 (6)	131 (3)
C4—H4···*Cg*1^iii^	0.93	2.99	3.6957	134
C7—H7···*Cg*1^iv^	0.93	2.99	3.6980	134

Symmetry codes: (ii) *x*−1/2, *y*+1/2, −*z*+1/2; (iii) −*x*+1/2, *y*−1/2, *z*; (iv) −*x*−1/2, *y*−3/2, *z*.
