# Supplementary figures and images for: Crystal structure of bis­(4-meth­oxy­phenyl) malonate
Source: Acta Crystallogr E Crystallogr Commun. 2015 Apr 22;71(Pt 5):o330–1. doi: 10.1107/S2056989015006891 (PMC4420049; doi:10.1107/S2056989015006891)

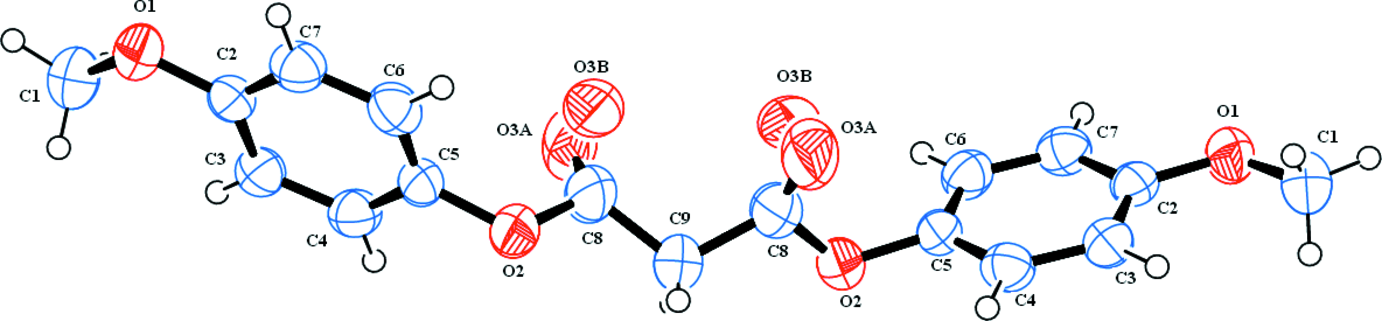

Supplement: Supplementary file 3 [file e-71-0o330-fig1.tif]

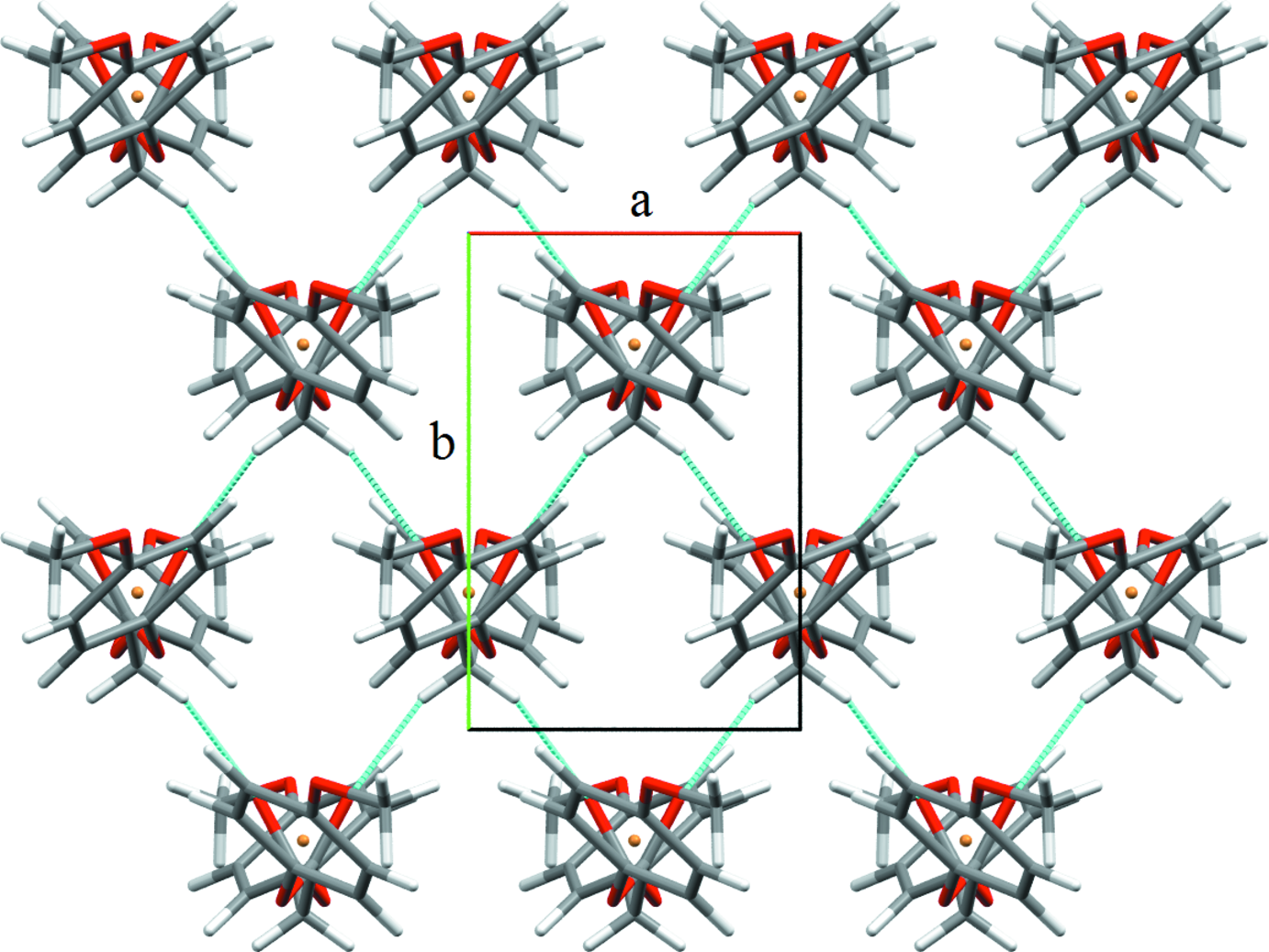

Supplement: Supplementary file 4 [file e-71-0o330-fig2.tif]

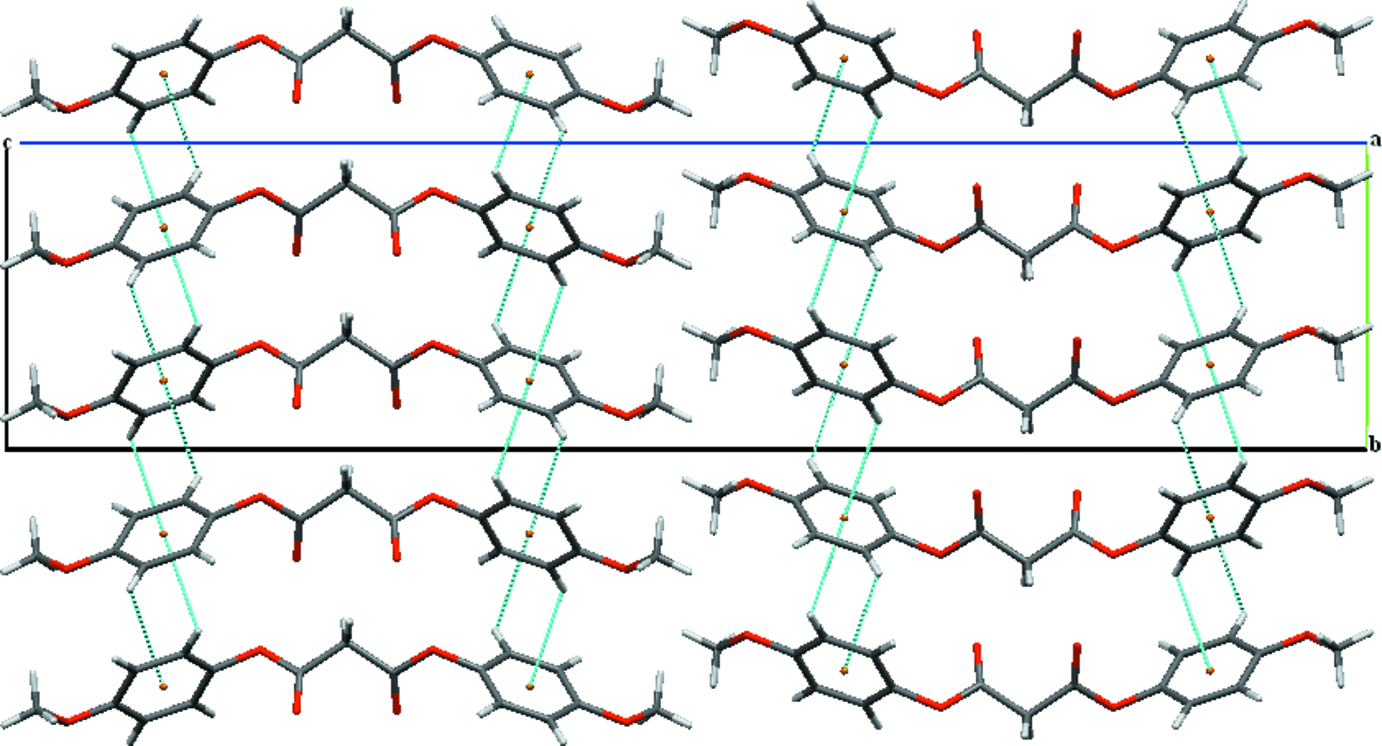

Supplement: Supplementary file 5 [file e-71-0o330-fig3.tif]
